# MENTAL HEALTH IMPACT OF DISCRIMINATION: THE PROTECTIVE FUNCTION OF SOCIAL CAPITAL IN DIVERSE GROUPS OF OLDER ADULTS

**DOI:** 10.1093/geroni/igac059.1326

**Published:** 2022-12-20

**Authors:** Mengzhao Yan, Yuri Jang, Kathleen Wilber

**Affiliations:** University of Southern California, Los Angeles, California, United States; University of Southern California, Los Angeles, California, United States; University of Southern California, Los Angeles, California, United States

## Abstract

Discrimination occurs in complex social contexts leading to various levels and types of outcomes. Although the negative health impact of discrimination is well-documented, there is a need to investigate patterns among discrimination, social factors, and health outcomes in diverse racial/ethnic groups of older adults to inform interventions. For example, social capital, such as social cohesion, social ties, and safety, is anticipated to be directly associated with mental health and also to modify the impact of discrimination. In the present study, we examined (1) racial/ethnic differences in perceived discrimination, social capital, and depressive symptoms and (2) the direct effect of perceived discrimination and social capital, as well as their interactions, on depressive symptoms among different racial/ethnic groups. Data were drawn from the National Social Life, Health, and Aging Project (NSHAP) Round 3 (2,988 non-Hispanic Whites, 719 non-Hispanic Blacks, and 499 Hispanics; 68 mean age). Compared to non-Hispanic Whites, non-Hispanic Blacks had a significantly higher level of perceived discrimination, lower social capital, and more depressive symptoms. Findings from multivariate linear regression models demonstrated that, in all racial/ethnic groups, frequent experiences of discrimination and low levels of social capital were associated with increased symptoms of depression. A significant interaction between discrimination and social cohesion was observed in non-Hispanic Whites and Hispanics. In both groups, the negative impact of discrimination was lower among those with higher levels of social cohesion. Our findings support efforts such as improving well-being for older adults by promoting age-friendly communities to build greater social cohesion.

